# Case report: Pembrolizumab as an alternative to atezolizumab following a severe infusion reaction

**DOI:** 10.3389/fonc.2024.1273043

**Published:** 2024-03-04

**Authors:** Seung Hyuk Lee, Hyeon Jong Kim, Hyun Jin Bang, Su Ji Park, Ji Eun Yu, Seung Woo Jeong, Woo Kyun Bae

**Affiliations:** ^1^ Division of Hematology-Oncology, Department of Internal Medicine, Chonnam National University Medical School and Hwasun Hospital, Hwasun, Republic of Korea; ^2^ Department of Pharmacy, Chonnam National University Bitgoeul Hospital, Gwangju, Republic of Korea; ^3^ Division of Allergy and Clinical Immunology, Department of Internal Medicine, Chonnam National University Hospital, Gwangju, Republic of Korea; ^4^ Chonnam National University College of Medicine, Hwasun, Republic of Korea

**Keywords:** immune-checkpoint inhibitors (ICIs), atezolizumab, pembrolizumab, infusion reaction, hypersensitivity, immune-related adverse drug reactions (ADRs)

## Abstract

The emergence of immune-checkpoint inhibitors (ICIs) has revolutionized the field of oncology, providing promising results in various malignancies. However, ICIs can sometimes lead to severe injection reactions, requiring alternative treatment options. In this case report, we introduce a case of a severe infusion reaction induced by atezolizumab. After atezolizumab infusion, the patient experienced symptoms that were suggestive of anaphylactic shock, including chest tightness, low blood pressure, and loss of consciousness, all of which were restored by immediate administration of steroid, antihistamine, and epinephrine. When selecting a new ICI, we were concerned about cross-reactivity with atezolizumab. As such, we conducted a skin test to establish the underlying mechanism of the previous reaction to atezolizumab infusion, the results of which were highly suggestive of Ig-E-mediated hypersensitivity. The skin test for pembrolizumab, another ICI, was negative. Therefore, we replaced atezolizumab with pembrolizumab, and the infusion proceeded safely. To date, the patient has undergone 13 cycles of pembrolizumab, and the disease has remained stable. This case demonstrates that patients who exhibit severe injection reactions to ICIs can continue treatment safely, without cross-reactions, with alternative ICIs. This case will help provide patients who have experienced drug-related hypersensitivity reactions with a choice to use alternative ICIs, thus expanding their options for chemotherapy.

## Introduction

Immunotherapy has revolutionized the treatment landscape for various malignancies, with immune-checkpoint inhibitors (ICIs) emerging as a promising class of therapeutics (1). ICIs function by blocking immune checkpoints, thereby enhancing the immune system’s ability to target cancer cells (1). Although each ICI has a unique molecular target, they share a common mechanism of action and may exhibit overlapping adverse effects (1).

Atezolizumab, a monoclonal antibody targeting programmed death-ligand 1 (PD-L1), has emerged as a promising immunotherapeutic agent for the treatment of various malignancies (2–4). Although atezolizumab has demonstrated significant efficacy in boosting the immune system’s ability to combat cancer, its use is occasionally associated with infusion reactions, posing challenges with clinical administration (5).

In the context of cancer immunotherapy, the immune-related adverse drug reactions (ADRs) are an important consideration. The pathophysiology of ICI-related ADRs is complex and involves T-cell activation against self-antigens, leading to inflammatory responses in the affected tissues. However, Infusion reactions related to ICIs are typically non-IgE-mediated hypersensitivity reactions that can occur during or shortly after the administration of these agents. So it is imperative to discern between infusion reactions specific to ICIs and drug hypersensitivity reactions, as the clinical management and potential implications for ongoing cancer treatment significantly differ between the two (1, 2).

Treatment for metastatic urothelial cancer typically includes chemotherapy as a first-line treatment, often with a combination of drugs such as cisplatin and gemcitabine. In recent years, immunotherapy involves drugs like pembrolizumab or atezolizumab has become a vital part of the treatment for metastatic urothelial cancer, especially for patients who don’t respond well to chemotherapy (3).

In this case report, we present the clinical course of a patient with metastatic ureter cancer who received atezolizumab as a primary treatment. The patient had a serious and unexpected infusion reaction similar to anaphylaxis during the first injection of atezolizumab. Despite concerns surrounding cross-reactions to other ICIs, pembrolizumab was selected as an alternative and was infused with no adverse effects.

This case report aims to provide a comprehensive review of infusion reactions linked to atezolizumab, including their clinical manifestations, underlying mechanisms, management strategies, and implications for patient care. This unprecedented scenario provides an opportunity to explore the safety, effectiveness, and potential implications of using pembrolizumab as an alternative to atezolizumab, which avoids the drug reaction that occurred during the first infusion. Furthermore, by reporting this case, we aim to contribute to the growing body of literature surrounding immune-related adverse events associated with ICIs.

## Case description

A 74-year old man with a past medical history of hypertension underwent nephrectomy and ureterectomy for a right ureter tumor on May 23, 2017. Adjuvant chemotherapy was recommended, but the patient refused and requested regular follow-up only. After 4 years, the patient developed abdominal lymph node metastases and received systemic chemotherapy. The patient was administered a chemotherapy regimen consisting of gemcitabine, dosed at 1000mg/m², and cisplatin, dosed at 35mg/m². This regimen was scheduled over a three-week cycle, with the patient receiving treatment during the first two weeks and having the third week as a rest period. After seven cycles of chemotherapy, the recurred lesion progressed, and the chemo-agent was changed to atezolizumab, a PD-L1 inhibitor. Ten minutes after the first infusion of atezolizumab, the patient complained of dyspnea and itching, and displayed hypotension with systolic blood pressure decreasing to 40 mmHg. Subsequently, his oxygen saturation decreased to 80% and he lost consciousness. The level of consciousness was assessed as Glasgow coma scale 5. The patient was immediately administered antihistamine, steroid, epinephrine, and fluid to treat the hypersensitivity reaction. Afterwards, blood pressure and consciousness recovered within minutes. As a result, consciousness was restored, and vital signs stabilized. Causality assessments suggested that the event met the WHO-UMC causality assessment as terms of “probable” (4)., with a Naranjo’s score of 7 (5).

Given the severity of the reaction to atezolizumab, it was decided to cease further administration; instead, salvage radiation therapy was performed on the recurred lymph node lesions. During the period 22/3/10-3/31, salvage radiation therapy was applied 25 times to the ureter and surrounding lymph nodes, for a total of 3200cGy. Initially, radiation therapy provided a stable response, but the metastatic lymph node lesions worsened again 5 months after the end of radiation treatment. We discussed with the patient whether to re-administer systemic chemotherapy or try another ICI, pembrolizumab, and the patient expressed that they wished to try pembrolizumab. Given the possibility that the severe infusion reaction that occurred after the administration of atezolizumab was type 1 hypersensitivity, and the possibility that a new ICI would cross-react with atezolizumab, we decided to conduct a skin test on both ICIs.

## Diagnostic assessment and details of the therapeutic intervention, follow-up, and outcomes

**Figure 1:**
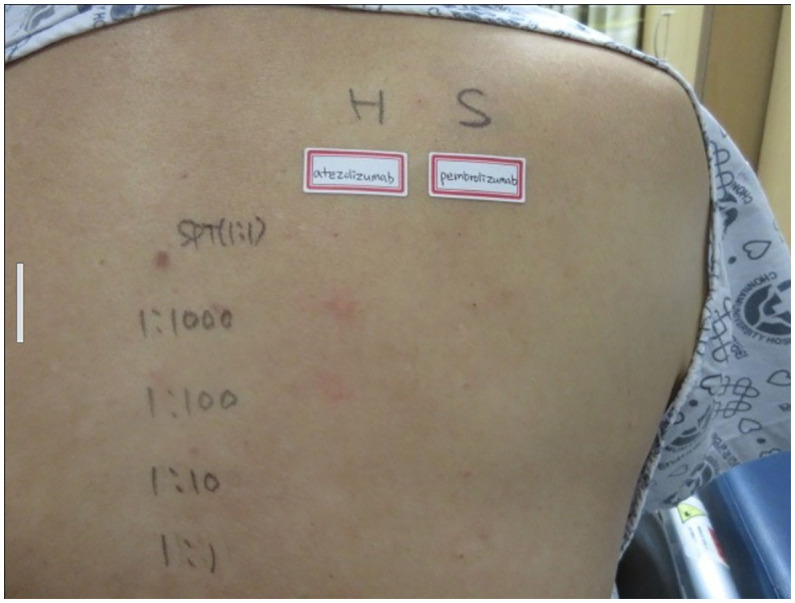
Skin prick test: negative for atezolizumab and pembrolizumab. Intra-dermal test: positive at 0.06 mg/mL and 0.6 mg/mL of atezolizumab (IDT; 0.06 mg/mL; wheal, 3.8 × 3.5; flare, 7 × 7 and 0.6 mg/mL; wheal, 5.3 × 4.9; flare, 12 × 8), and negative for pembrolizumab.

**Figure 2:**
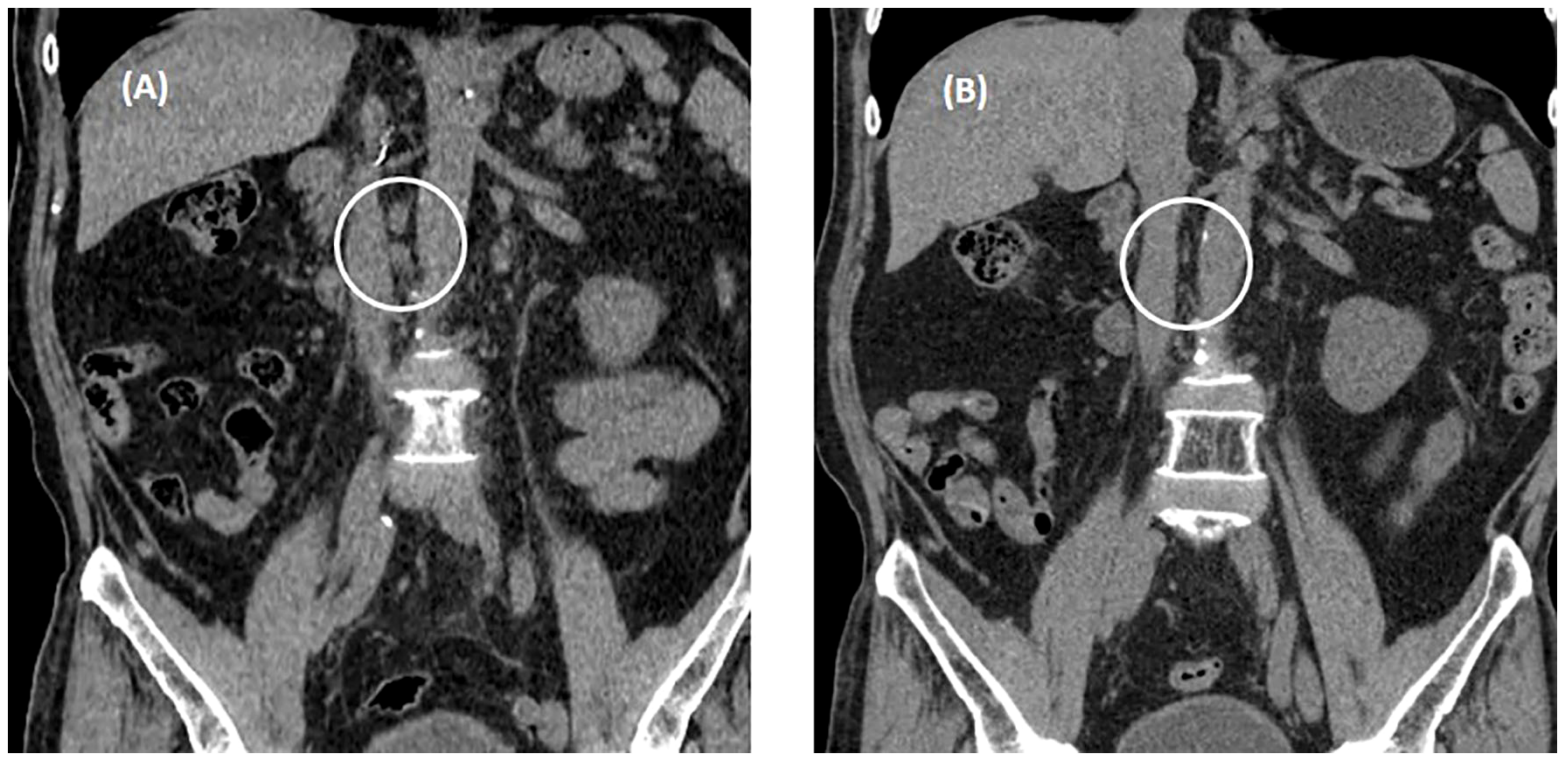
Abdominal non-contrast computed tomography after three cycles of pembrolizumab. **(A)** Enlarged metastatic lymphadenopathies in aorto-caval, and para-aortic on CT conducted August 22, 2022. **(B)** A partial response was observed on restaging CT conducted October 18, 2022. White circle means "metastatic LNs".

The drug dose used in the skin test was determined by referring to a previous study (6, 7). A positive reaction in the skin test was defined according to the criteria recommended by the American Allergy Society (8): Positive skin test, development of a wheal that is at least 3 mm greater than that observed with the negative control for prick/puncture; or intradermal test (IDT) accompanied by a flare > 5 mm. The skin prick test with atezolizumab at a concentration of 60 mg/mL was negative, and the intradermal test was positive at 0.06 mg/mL and 0.6 mg/mL (IDT, 0.06 mg/mL; wheal, 3.8 × 3.5; flare, 7 × 7 and 0.6 mg/mL; wheal, 5.3 × 4.9; flare, 12 × 8) (**Figure 1**). The skin prick test (25 mg/ml) and intradermal (0.25 mg/ml) test were performed with pembrolizumab, and both were negative (**Figure 1**). Therefore, we decided to carefully infuse pembrolizumab, without steroid or antihistamine injection, and the first injection was completed safely, without any hypersensitivity-related symptoms. We introduced Keytruda at 3-week intervals and conducted restaging CT scans every 3 months. After the 11th chemotherapy session, a partial response was still maintained (**Figure 2**). After the 20th infusion, CT scan showed disease progression, leading to the discontinuation of Keytruda.

## Discussion

As various types of ICIs continue to develop, interest in incidence, mechanisms, preventions of immune-related ADRs are also being actively growing. Previous autoimmune disease, genetic predisposition, combination therapy, using ICIs in combination with other therapies can increase the risk of ADRs. Some evidence suggests that the type and stage of cancer may affect the likelihood of experiencing immune-related ADRs. For instance, melanoma patients treated with CTLA-4 inhibitors may experience different ADRs compared to those with lung cancer treated with PD-1/PD-L1 inhibitors. Research has indicated that there may be sex-based differences in the incidence and severity of immune-related ADRs. However, the data is not entirely conclusive, and more research is needed to understand these differences fully. Elderly patients may have a different risk profile for ADRs due to age-related changes in the immune system and a higher likelihood of comorbidities (9).

Most anticancer agents, including ICIs, carry a risk of adverse drug reactions, especially infusion reactions, with several reports of infusion reactions after using ICIs (10, 11). Infusion reactions can be classified as type 1 hypersensitivity reactions (immune-mediated adverse reaction) and non-allergic reactions, such as cytokine-release syndrome (CRS) (1, 12). Regardless of whether the reaction is allergic or non-allergic, the clinical manifestations are the same and require accurate assessment and acute management (1).

Currently, there is no unified consensus on whether the mechanism of infusion reactions caused by monoclonal antibodies, including ICIs, is immune-mediated or a symptom of CRS (1, 12). However, it is generally accepted that the culprit drug should be discontinued if a serious infusion reaction is observed. Clinically, anaphylaxis is diagnosed by measuring the serum tryptase level, conducting skin tests, and measuring the serum allergen-specific IgE levels to identify the allergen (13). Blood samples for the measurement of tryptase should be obtained 15 min to 3 h after symptom onset. In this case, the tryptase level was measured 4 h after the onset of symptoms and showed a value of 17.4, which is above the normal range (1–11.4 ng/mL).

Severe infusion reactions with atezolizumab are rare, but a few related cases have been reported (6, 14). Although successful desensitization with atezolizumab has been reported (6), re-administration after severe infusion reactions should be carefully considered. There has been no specific evidence reported that suggests anti-PD-L1 ICIs are more likely to cause anaphylactic or immune-related adverse drug reactions (ADRs) compared to other ICIs. The safety profiles of ICIs can vary due to their different molecular structures and mechanisms of action. Anti-PD1 and anti-PD-L1 antibodies differ in their target interactions; for instance, anti-PD1 antibodies block the binding of PD-1 to both of its ligands, PD-L1 and PD-L2, while anti-PD-L1 antibodies specifically block the interaction between PD-1 and PD-L1. These differences could theoretically influence the immunogenicity of the drugs and result in different safety profiles. However, the clinical significance of these differences in terms of ADRs, including anaphylactic reactions, is still being studied and is not fully understood (15).

In this case, we determined whether the infusion reaction that occurred after the use of atezolizumab was IgE-mediated hypersensitivity or CRS. The results of the skin prick test and serum tryptase levels were highly indicative of type 1 hypersensitivity. Typically, type 1 hypersensitivity requires sensitization to a specific antigen, but in this case, the patient had not been previously exposed to atezolizumab. As an example of cross-reactivity, it is possible that this patient was sensitized to a drug or food with a similar epitope to atezolizumab. In the context of alpha-gal syndrome, anaphylaxis can be triggered when an individual who has been sensitized to a sugar molecule called alpha-gal, found in red meat, receives their first dose of the drug cetuximab. Cetuximab contains the alpha-gal molecule, and exposure to it can prompt an allergic reaction in those who have developed sensitivity. The sensitization to alpha-gal can lead to an immune response upon subsequent exposure to it through certain medications, resulting in anaphylaxis (16). However, it is difficult to exclude the possibility that the skin prick test or elevated serum tryptase directly activated mast cells or was a false positive result.

Although the burden of side effects related to cytotoxic drugs has been alleviated with the introduction of ICIs, cases of severe infusion reactions due to ICIs are still reported occasionally (14, 17, 18). Understanding the mechanisms underlying these infusion reactions is critical to effectively manage and mitigate associated risks, but elucidating the hidden pathways of immune responses remains a considerable challenge for physicians. Therefore, as an appropriate alternative, the introduction of another ICI should be carefully considered. This will require individualized treatment decisions based on patient characteristics, including tumor type, previous therapy, and potential for cross-reactivity between ICIs. Strategies for monitoring and managing infusion reactions and other adverse events associated with ICIs are considered worthy of discussion.

## Data availability statement

The raw data supporting the conclusions of this article will be made available by the authors, without undue reservation.

## Ethics statement

Ethical approval was not required for the studies on humans in accordance with the local legislation and institutional requirements because only commercially available established cell lines were used. Written informed consent was obtained from the individual(s) for the publication of any potentially identifiable images or data included in this article.

## Author contributions

SL: Writing – original draft, Writing – review & editing, Conceptualization. HK: Writing – original draft. HB: Writing – original draft. SP: Data curation, Writing – original draft. JY: Conceptualization, Data curation, Writing – original draft. SJ: Investigation, Writing – original draft. WB: Conceptualization, Supervision, Writing – review & editing.
